# Bilateral spontaneous thrombosis of the pampiniform plexus mimicking incarcerated inguinal hernia: case report of a rare condition and literature review

**DOI:** 10.1186/s40792-020-00810-3

**Published:** 2020-03-05

**Authors:** Sabyasachi Bakshi

**Affiliations:** 1 Department of General Surgery, BSMCH, Bankura, India; 2 Hooghly, 712103 West Bengal India

**Keywords:** Pampiniform plexus, Incarcerated inguinal hernia, Thrombosis

## Abstract

**Background:**

Pampiniform plexus thrombosis is a very rare disease (only less than 25 published cases are available till date), and it is a diagnostic dilemma. The present case is an unusual condition of an elderly gentleman who was finally diagnosed as a case of spontaneous thrombosis of bilateral pampiniform plexus and was managed conservatively. Literature was reviewed to explore potential etiologies and therapeutic strategies.

**Case presentation:**

A 65-year-old afebrile gentleman, laborer (in brick industry), and non-smoker with no previous major health problems was admitted with swelling in the bilateral inguinal region. The swelling had started one and half months ago. He had developed severe pain over the swelling for last 1 day with tenderness and indurations. Neither he had history of previous surgeries, chronic cough, dysuria, prostatism, and trauma nor he presented any thrombogenic factors. There was no history of vomiting, abdominal pain, and obstipation. Physical examination revealed normotensive person with BMI of 22.5, was significant only for one tender, movable, and firm to hard 10 cm × 3 cm mass extending from the left deep inguinal ring up to the upper pole of the testis in the scrotum. Another 5 cm × 3 cm mass of similar characteristics was found extending from deep inguinal ring up to the root of the scrotum on right side. The testes and prostate were normal on palpation.

On the contrary to preoperative USG, which clinched suspicion of incarcerated inguinal hernia, a thrombosed pampiniform plexus without any evidence of hernia sac was found on the left side during inguino-scrotal exploration. Wound was closed without doing any further procedure. Contralateral inguino-scrotal exploration was spared considering same nature of disease. Postoperative Doppler ultrasonography confirmed the diagnosis of bilateral thrombosed pampiniform plexus. MDCT of whole abdomen revealed no abnormality other than bilateral spermatic cord thrombosis. Blood thrombophilia screening came normal. The subject had an uneventful postoperative hospital course. With 2 years of follow-up, the gentleman is doing well, remaining asymptomatic and had returned to his usual life.

**Conclusions:**

Due to extreme rarity, spontaneous thrombosis of the pampiniform plexus may be a diagnostic dilemma and requires a high index of suspicion. Doppler ultrasound is the initial investigation of choice. In the absence of other concomitant disease, beginning the treatment conservatively instead of excising the thrombosed segment is more suitable.

## Background

Spontaneous pampiniform plexus thrombosis is a diagnostic dilemma, and it is a very rare condition. Less than 25 cases of spontaneous thrombosis have been published in the literature till date [1]. Acute inguino-scrotal or testicular painful swelling is the usual clinical presentation [1], and commonly left spermatic cord gets involved [2]. Preoperatively, it may be misdiagnosed due to its non-specific presentation and as it is clinically indistinguishable from many other inguinal conditions. The present case is an unusual condition of an elderly gentleman, with idiopathic spontaneous thrombosis of bilateral pampiniform plexus. The present report is also the first ever reported case of bilateral pampiniform plexus thrombosis. Literature was also reviewed to explore potential etiologies and therapeutic strategies to manage this extremely rare condition.

## Case presentation

A 65-year-old afebrile gentleman, laborer (in brick industry) and non-smoker with no previous major health problems, was admitted for painful swelling in the bilateral inguino-scrotal region. The swelling had started one and half months ago in the bilateral inguinal region, and later, it gradually involved the upper part of the scrotum bilaterally. The swelling was small initially, but gradually attained presenting size in the last 4–5 days. The swelling did not reduce on lying down, but it used to get prominent in standing position. Initially, there was mild dragging and aching pain over the swelling, but the pain was increased and became severe since 1 day with tenderness and indurations. There was no history of vomiting, abdominal pain, dysuria, and obstipation. Neither he had history of previous surgery, chronic cough, prostatism, and trauma nor he presented any thrombogenic factors.

Physical examination revealed normotensive person with BMI of 22.5, was significant only for one left sided elongated, tender, movable, and firm to hard 10 cm (vertical) × 3 cm (horizontal) mass (above the crease of groin and medial to pubic tubercle). It was extending from the left deep inguinal ring up to the upper pole of the testis in the scrotum. Local temperature over the swelling was raised with mild erythama. There was no visible or palpable cough impulse. “Get above the swelling” was not possible. As the swelling was irreducible, the deep ring occlusion test could not be performed. Dull note was found on percussion with no audible gurgling sound. Another 5 cm (vertical) × 3 cm (horizontal) mass of similar character was found extending from the right deep inguinal ring up to the root of the scrotum. Both the testes were normally positioned in the scrotum, but the left one was mildly swollen. Prostate size and penile position were normal. Umbilicus was in normal position in scaphoid abdomen without any tenderness, visible peristalsis, or palpable mass.

**Fig. 1:**
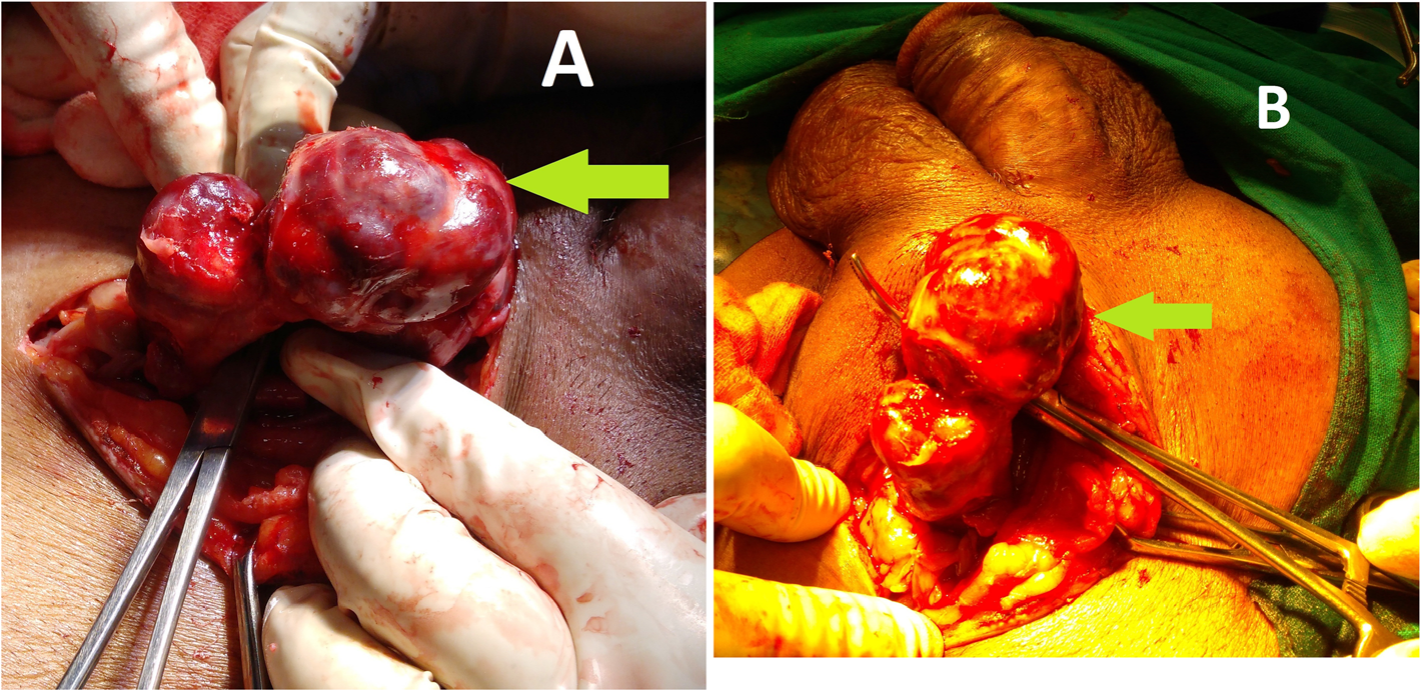
**a**, **b** Intra-operative photographs. Green arrows show thrombosed left pampiniform plexus

**Fig. 2:**
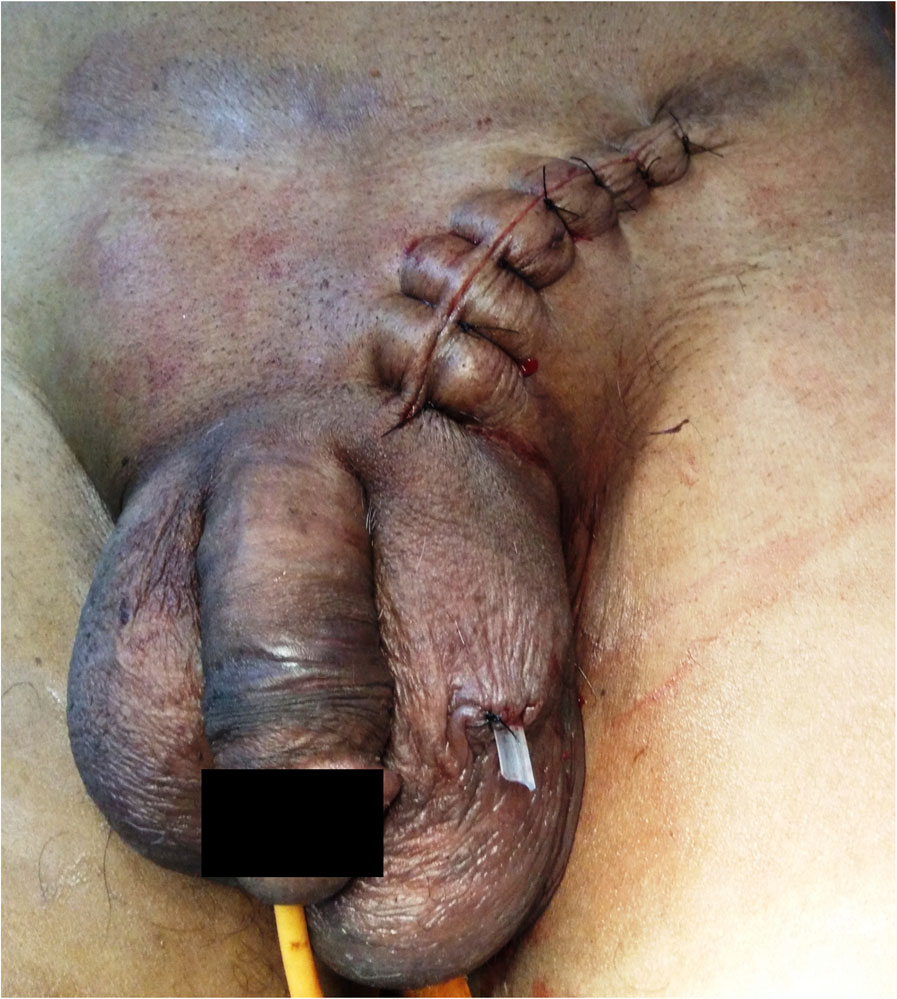
Immediate post-exploration photograph shows indurations and swelling of both inguinal region

Contrary to pre-operative grayscale USG finding (hernia containing tubular loops), which clinched the suspicion of incarcerated inguinal hernia, on exploration of left inguino-scrotal region under spinal anesthesia, left spermatic cord was found to be thick, multi-lobulated, blackish-red colored, tubular mass of firm to hard consistency (Fig. 1a, b). This was thrombosed pampiniform plexus without any evidence of hernia sac, and the testis was found to be mildly congested. Decision of no further intervention was taken. Wound was closed. Contralateral inguino-scrotal exploration was spared considering the same nature of disease (Fig. 2).

**Fig. 3:**
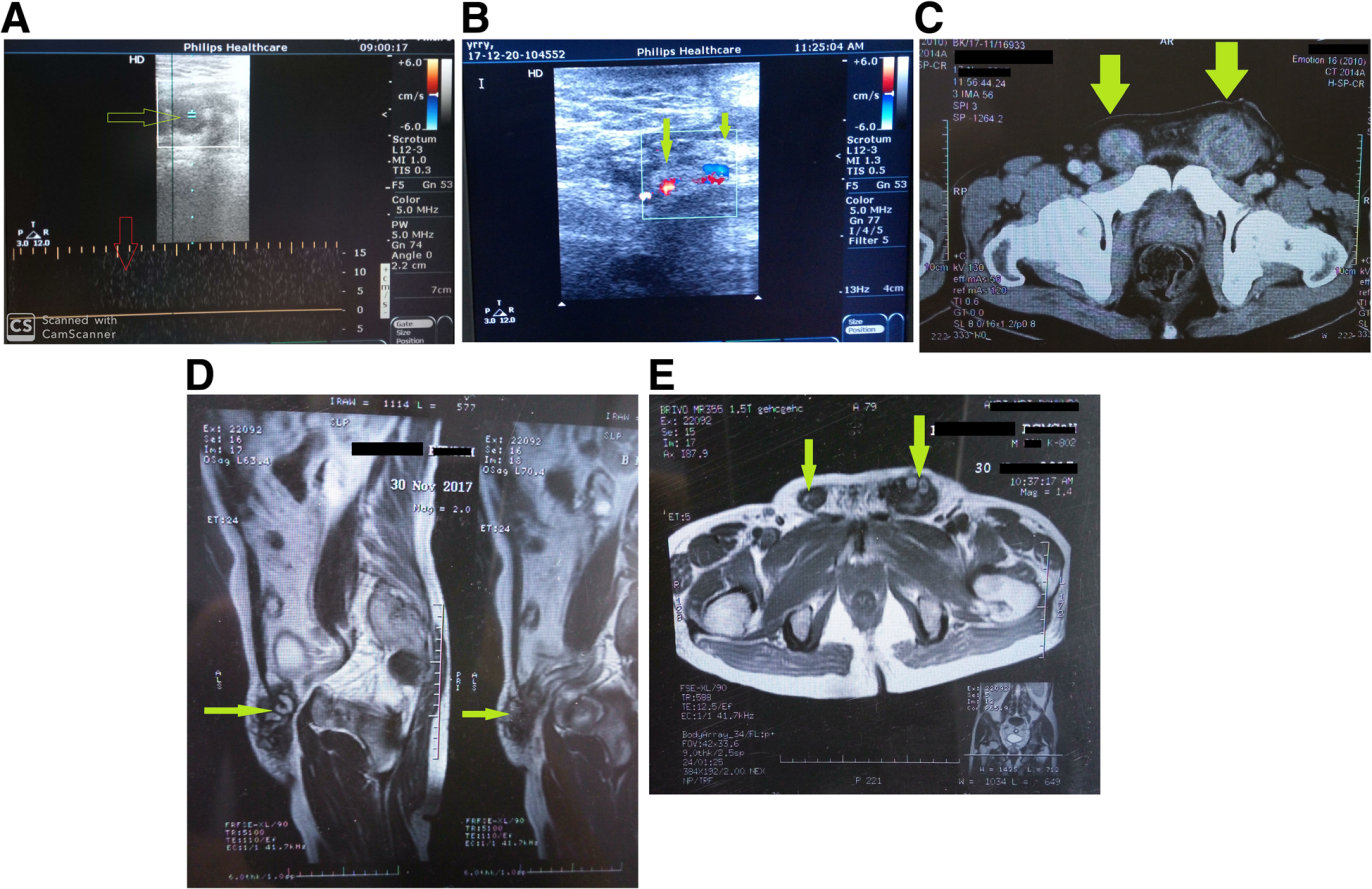
**a** Grayscale ultrasonography demonstrate dilated, non-compressible, thrombosed tubular venous structure with increased wall thickness within the left spermatic cord. Within this tubular structure, focal echoes that belong to thrombus (green arrow) can be seen, but no vascular flow curve can be seen (red arrow). **b** On color Doppler ultrasound, no filling with the color was seen in the lumen of this vein within the left spermatic cord. On Doppler ultrasound, filling was seen within the neighboring arterial structure (green arrows), but not within the vein. **c** Computed tomography scan showing grossly distended and thrombosed spermatic veins (green arrows). **d** Sagittal section and **e** transverse section: fat-compressed axial T1 magnetic resonance images demonstrated thrombosed tubular venous structure (green arrows) with increased wall thickness and focal diameter increase within the bilateral spermatic cord. Within this venous structure, intraluminal signal intensity was increased

**Fig. 4:**
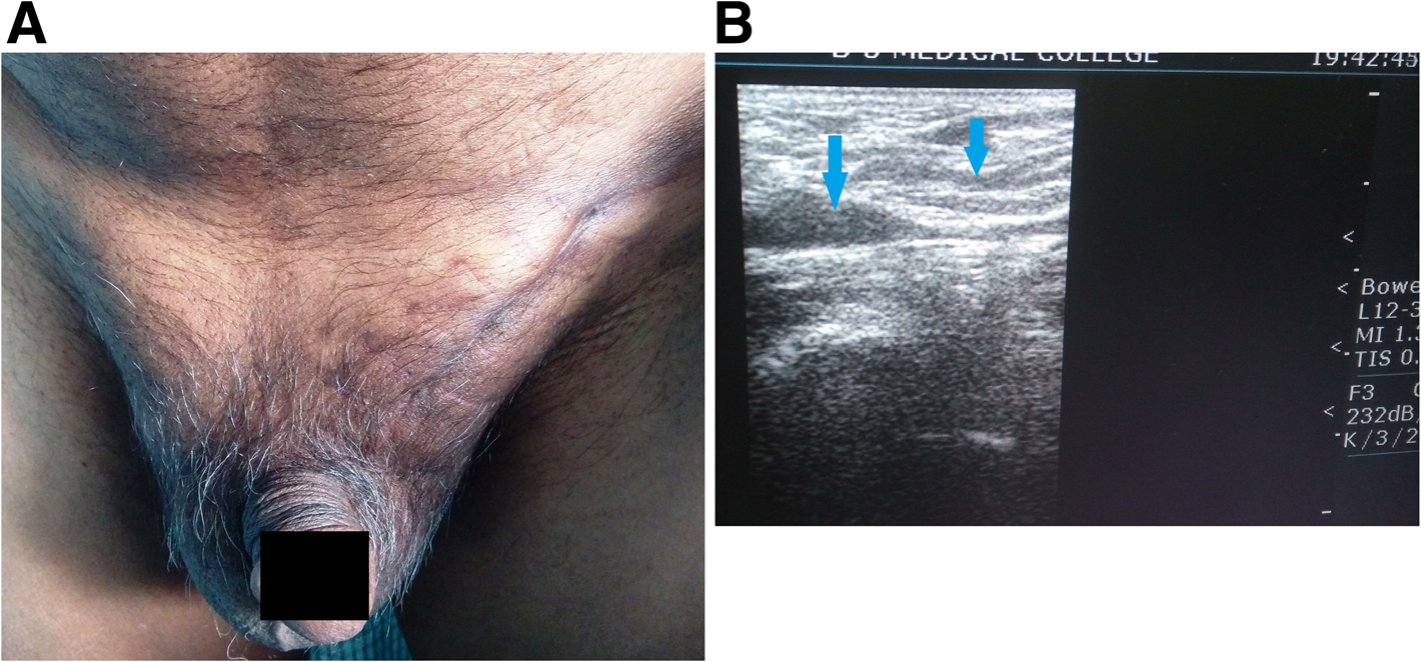
**a** Photograph of inguino-scrotal region on follow-up at 4 months. **b** USG of left spermatic cord shows no thrombus, and full compressibility was noted on 4 months follow-up

Post-operative period was uneventful. The patient was put on anti-inflammatory drugs. Oral feeding was started from the next day, and early ambulation was encouraged. The swelling and pain started to get reduced gradually. There was no episode of shortness of breath or chest pain, tachycardia, and tachypnea in post-op period. BP was normal throughout the post-operative period. There was no development of calf tenderness. Post-operative ultrasonography with color Doppler study (Fig. 3a, b) confirmed the diagnosis of bilateral thrombosed pampiniform plexus, showing hyperechoic soft tissue mass lesions in bilateral spermatic cords. Very few vessels were seen within the mass with colored flow. Bilateral testis was normal. MDCT and MRI scan of the whole abdomen (Fig. 3c, d, e) revealed no abnormality other than bilateral spermatic cord thrombosis. Blood thrombophilia screening (factor V Leiden, prothrombin time, antithrombin assay, protein C and S, lupus anticoagulant, anticardiolipin antibody) came normal. ECG and urine analysis were normal. There was no surgical site infection. The patient was discharged in a stable condition after 7 days. The subject, with 2 years follow-up, is doing well, remaining asymptomatic and had returned to his usual life (Fig. 4a, b). He was advised regular check-up in surgical out-patients’ department.

## Discussions

Spermatic vein thrombosis is an unexpected finding in the differential diagnosis of acutely painful inguino-scrotal region [3]. Most of these cases are initially tried surgically as if they had an incarcerated inguinal hernia [4]. Additionally, epididymitis, spermatic cord disease (such as torsion), or benign and malignant tumors of spermatic cord should be kept in mind in the differential diagnosis [5]. Hashimoto and Vibeto [2] noted that there was a preponderance of left-sided presentations with presumed, shared anatomical factors which can also predispose to varicocele formation. Right spermatic vein thrombosis is an important clinical sign to do detailed research at the renal hilus level or in the retroperitoneal region to rule out renal/retroperitoneal tumors with renal vein, vena cava thrombosis. The present report is also the first ever reported case of bilateral pampiniform plexus thrombosis. The author also studied the characteristics of all available cases [6] in chronological order (Table 1) which revealed (Table 2 ):

**Table 1:** Description of characteristics of all available cases, reported till day, in chronological order

Serial no.	Age (years)	Location of lesion	Onset of pain	Predisposing factors	Diagnosis (provisional)	Investigations	Management	Publication year and author
1	NA	Left	NA	NA	Orchitis	None	NA	1903, Senn [7]
2	NA	NA	“Sudden”	None	Thrombosis	None	Excision	1904, Senn [8]
3	41	Left	5 weeks	None	Orchitis	None	Orchidectomy	1935, Mc Gavin [9]
4	57	Left	4 weeks	None	Orchitis	None	Orchidectomy	1935, Mc Gavin [9]
5	27	Left	16 h	None	NA	None	Vein biopsy	1977, Anseline [10]
6	7	Left	NA	None	NA	Venography	Exploration	1980, Coolsaet and Weinberg [11]
7	10	Left	NA	None	Thrombosis	Venography	NSAID	1980, Coolsaet and Weinberg [11]
8	15	Left	11 days	Walking	NA	None	Excision	1980, Coolsaet and Weinberg [11]
9	33	Left	10 days	None	Incarcerated hernia	IVP	Excision	1981, Vincent and Bokinsky [12]
10	44	Right	“Hours”	Playing sports	Inguinal mass	None	Excision	1981, Rothman [13]
11	33	Left	NA	Varicocele	NA	None	Excision	1985, Roach et al. [14]
12	42	Contralateral	1 week	None	Incarcerated hernia	IVP, cavogram CT scan	Excision	1985, Roach et al. [14]
13	23	Left	“Hours”	Heavy weight lifting	Incarcerated hernia	Doppler USG	Excision	1990, Isenberg et al. [15]
14	19	Left	“Hours”	Vigorous exercise	Incarcerated hernia	None	Excision	1993, Gleason et al. [16]
15	27	Left	2–3 h	Heavy weight lifting	Incarcerated hernia	None	Exploration	2006, Hashimoto et al. [2]
16	33	Left	3 days	Cycling	Thrombosis	Doppler USG	NSAID	2009, Doerfler et al. [17]
17	NA	Contralateral	NA	NA	NA	NA	NA	2010, Kayes et al. [18]
18	28	Left	14 days	Nutcracker syndrome	NA	Doppler USG, CT scan	Excision	2014, Mallat et al. [19]
19	43	Right	2 days	Absence IVC, mutation factor V Leiden	NA	Doppler USG, CT scan	Anticoagulation	2015, Chi and Hairston [20]
20	39	Contralateral	3 days	Infection protein C deficiency	Thrombosis	Doppler USG, CT Scan	Antibiotics anticoagulant	2018, Kamel et al. [6]
21 (present case)	65	Bilateral	1 day	Heavy weight lifting	Incarcerated hernia	Doppler USG, MRI, CT scan blood test	NSAID	2020, Bakshi S

*NA* no available information, *IVP* intra-venous pyelogram, *CT scan* computed tomography scan, *IVC* inferior vena cava

**Table 2:** Comparative characteistics of present study

Parameters	Findings after literature review	Findings of the present case
Age at presentation	Mean age was found 32.27 years (range 7–65 years)	Present case is the eldest of all reported subjects till date
Location (side)	Left sided in 70% cases, 25% in right side	Present case is the only reported case of bilateral thrombosis
Duration of pain	Varied duration. Ranges from hours to 5 weeks	In the present case, mild dragging pain started 6 weeks ago
Predisposing factors	Majority reported heavy physical works	Subject in the present case was also an active physical labor
Initial diagnosis	Majority was diagnosed preoperatively as incarcerated inguinal hernia	Present case was also diagnosed as incarcerated inguinal hernia in the emergency department
Primary investigation and management	USG Doppler flow study confirmed majority of the cases, and majority were managed by surgical excision	USG Doppler confirmed diagnosis. But the case was managed conservatively

In the etiology of isolated spermatic vein thrombosis, there are many possible predisposing factors, such as trauma to the vascular endothelium, slow venous flow, and hypercoagulability [21]. Kayes et al. reported that spontaneous vein thrombosis could be related to prolonged vigorous activity (e.g., heavy weight lifting, sports, physical training), tumors of the genitourinary tract, infections, trauma, inguinal hernia surgery, long-hour flights, and the use of some drugs [22]. An increase in intra-abdominal pressure linked to these activities may decrease flow within the gonadal venous systems which may be compounded by specific anatomical factors. Most notably, in keeping with a left-sided predominance of this condition, one must consider meso-aortic compression of the left renal and spermatic vein(s), also known as “nutcracker syndrome” [23]. Examination with Doppler ultrasound should be the first-line investigation, while others outlined in previous case reports include a thrombophilia screen [24], MDCT of the abdomen to rule out causes of venous obstruction, incarcerated hernia, or malignancy [12]. As spermatic vein thrombosis is clinically indistinguishable from many other groin conditions, computed tomographic angiography may help to reveal whether the thrombus extends beyond the external inguinal ring. It also helps to find the etiology, such as nutcracker syndrome especially in young male.

In the literature also, no report regarding recurrence was found after conservative management. There are no guidelines available for the management of this disease. Hashimoto and Vibeto reported that there is no need to excise the thrombosed plexus, as evidenced by the good results in their case [2]. Conservative management, including watchful observation and NSAID without anti-coagulation, is acceptable for thrombosis out of external inguinal ring (pampiniform plexus). Yoko Kyono et al. proposed surgical excision, and anticoagulation may prevent pulmonary embolism in deep-seated spermatic vein thrombus inside the external inguinal ring and extending to the nearby renal vein [25]. Though the management remains unclear, proximal extension of the thrombosis is the most significant indication for further investigation.

## Conclusions

Isolated spermatic vein thrombosis is a rare event and requires a high index of suspicion. Although present case is bilateral, spermatic vein thrombosis is almost always found at the left side. Doppler ultrasonographic examination is the procedure of choice in the diagnosis of the varicocele thrombosis with higher sensitivity and specificity. Exploratory surgical approach may be needed initially in the absence of Duplex study, to exclude an acutely infarcted testis or incarcerated hernia. But in the confirmed absence of other concomitant disease that necessitates urgent surgical intervention, beginning the treatment conservatively instead of excising the thrombosed segment is more suitable. Although conservative management, including watchful observation and NSAID without anti-coagulation, is acceptable for thrombosis out of external inguinal ring (pampiniform plexus), surgical excision and anticoagulation may prevent pulmonary embolism in deep-seated spermatic vein thrombus (proximal to the external inguinal ring) and extending to the nearby renal vein. Surgeons should be aware of this rare clinical entity for prompt management of potential morbidity.

## Data Availability

Presented within the manuscript. Please contact author for additional data requests.

## Abbreviations

BMI: Body mass index
BP: Blood pressure
ECG: Electrocardiography
MDCT: Multi-detector computed tomography
MRI: Magnetic resonance image
NSAID: Non-steroidal anti-inflammatory drugs
USG: Ultrasonography
