# Correction to: Bilateral spontaneous thrombosis of the pampiniform plexus mimicking incarcerated inguinal hernia: case report of a rare condition and literature review

**DOI:** 10.1186/s40792-020-00821-0

**Published:** 2020-03-23

**Authors:** Sabyasachi Bakshi

**Affiliations:** 1 Department of General Surgery, BSMCH, Bankura, India; 2 Hooghly, West Bengal 712103 India

**Correction to: Surg Case Rep**

**https://doi.org/10.1186/s40792-020-00810-3**

In the original publication of this article [1], there is a correction in Table 2. The revised Table 2 is shown below.

**Table 2:** Comparative characteristics of present study

Parameters	Findings after literature review	Findings of present case
Age at presentation	Mean age was found 32.27 years.(range 7–65 years)	Present case is the eldest of all reported subjects till date.
Location (side)	Left sided in 70% cases, 25% in right side.	Bilateral thrombosis were found at presentation.
Duration of pain	Varied duration. Ranges from hours to 5 weeks	In present case mild dragging pain started 6 weeks ago.
Predisposing factors	Majority reported heavy physical works.	Subject in present case was also a active physical labor
Initial diagnosis	Majority was diagnosed preoperatively as incarcerated inguinal hernia.	Present case was also diagnosed as incarcerated inguinal hernia in emergency department.
Primary Investigation and management	USG Doppler flow study confirmed majority of the cases and majority were managed by surgical excision.	USG Doppler confirmed diagnosis. But the case was managed conservatively.
